# Association Between Red Blood Cell Distribution Width and COVID-19 Severity in Delta Variant SARS-CoV-2 Infection

**DOI:** 10.3389/fmed.2022.837411

**Published:** 2022-02-21

**Authors:** Jianguo Zhang, Jianhui Hu, Xing Huang, Shixiang Fu, Daoyin Ding, Zhimin Tao

**Affiliations:** ^1^Department of Emergency Medicine, The Affiliated Hospital of Jiangsu University, Zhenjiang, China; ^2^Department of Laboratory Medicine, Zhenjiang Hospital Affiliated to Nanjing University of Chinese Medicine, Zhenjiang Hospital of Traditional Chinese Medicine, Zhenjiang, China; ^3^Center for Evidence-Based and Translational Medicine, Zhongnan Hospital of Wuhan University, Wuhan, China; ^4^Department of Hepatology, The Third People's Hospital of Yangzhou City, Yangzhou, China; ^5^Department of Critical Care Medicine, The First People's Hospital of Jiangxia District, Wuhan, China; ^6^Jiangsu Province Key Laboratory of Medical Science and Laboratory Medicine, School of Medicine, Jiangsu University, Zhenjiang, China

**Keywords:** COVID-19, SARS-CoV-2, delta variant, red blood cell distribution width, vaccination

## Abstract

Studies have discovered that wild-type SARS-CoV-2 infections are commonly linked to abnormalities in the hematological profiles of COVID-19 patients, one such abnormality being characterized by elevations in red blood cell distribution width (RDW). Whether this linkage reoccurs in delta variant SARS-CoV-2 infection remains unexamined. Here we compared baseline blood parameters in COVID-19 patients infected by wild type and its delta variant, respectively. Our results here point to that although the delta variant has shown increased virulence, transmissibility, and vaccine escape, it has a minimally negative impact on RDW values that were previously found prognostic for COVID-19 severity.

## Introduction

Twenty-one months into the coronavirus disease 2019 (COVID-19) pandemic, the world still faces the rolling waves of viral hits as public health systems have been continuously stressed by this devastating disease. According to the World Health Organization (WHO) epidemiological report, as of December 5, 2021, the confirmed cases of COVID-19 infections have exceeded 264 million, with a cumulative death toll of over 5 million (1). Insofar a dozen variants of severe acute respiratory syndrome coronavirus 2 (SARS-CoV-2) have been identified, including the predominant delta variant and the upsurging omicron (1). In the latest coronavirus flare-ups, rapid spreading of SARS-CoV-2 in the form of delta variant has placed several cities in China under local lockdown, including Nanjing and Yangzhou in Jiangsu Province.

Red blood cell (RBC) distribution width (RDW) measures the heterogeneity of erythrocyte volumes, as abnormally elevated RDW values indicate anisocytosis of RBCs in circulation, being prognostic in a wide spectrum of human disorders (septic infection, cardiovascular diseases, cancers, etc.) and all-cause mortality (2). Recently, heightened RDW upon hospitalization was found in a close association with increased mortality for COVID-19 patients (3–5). Nevertheless, whether the RDW value upon hospital admission can predict the severity and mortality of delta variant SARS-CoV-2 infection remains unexplored.

## Results

We here report a total of 677 COVID-19 patients admitted at separate centers in China, where a cohort of 341 patients was hospitalized in Wuhan, Hubei Province during January-April 2020, and a cohort of 336 patients was hospitalized in Yangzhou, Jiangsu Province on August 2021 where the infections were identified to be caused by delta variant of SARS-CoV-2 (6). Clinical characteristics centered on RBC features were elaborated and compared between two cohorts of COVID-19 patients, to distinguish its delta variant from wild-type SARS-CoV-2 in their infection profiles. Details regarding the patient procedure, vaccination status, and statistical analysis can be found in Supplementary Material.

Between the two cohorts, no difference was shown in the median age, gender ratio, and major comorbidities (7). Table 1 lists the baseline hematological parameters for COVID-19 patients admitted during the outbreak at Wuhan. Anemia was common, reflected by low RBC counts, and hemoglobin and hematocrits levels in more than half of the patients. Other RBC indices, including mean corpuscular volume (MCV), mean corpuscular hemoglobin (MCH), and mean corpuscular hemoglobin concentration (MCHC) showed abnormality in a moderate to substantial portion of patients. Particularly, RDW displayed raised levels in more than half of COVID-19 patients, demonstrating the augmented distortion in RBC morphology. All RBC characters (except MCV) were prognostic indicators for COVID-19 severity but not mortality, as their differences between intensive care unit (ICU) and non-ICU patients were statistically significant while there was no noticeable difference in RDW between deceased and survived patients in the ICU. This stands in agreement with our own and others' previous findings (8–10).

**Table 1:** Baseline hematological data were compared between non-ICU and ICU COVID-19 patients infected by wild-type SARS-CoV-2, and between survivors and non-survivors after transfer into ICU.

	**Normal**	**Non-ICU (*n* = 245)**	**ICU (*n* = 96)**	***p*-value**	**Survival (*n* = 36)**	**Non-survival (*n* = 60)**	***p*-value**
RBCs, ×10^12^/L	4.3–5.8	4.3 (3.9–4.6)	3.5 (2.9–4.1)	<0.0001	3.7 (2.9–4.1)	3.4 (2.9–4.2)	0.907
<4.3		125 (51.0%)	81 (84.4%)	<0.0001	32 (88.9%)	49 (81.7%)	0.399
Hemoglobin, g/L	130–175	128.0 (115.0–140.0)	106.5 (87.3–123.8)	<0.0001	109.0 (88.0–123.0)	105.0 (84.8–127.8)	0.820
<130		132 (53.9%)	75 (78.1%)	<0.0001	29 (80.6%)	46 (76.7%)	0.655
HCT, %	40–50	37.5 (34.3–40.8)	31.9 (26.5–37.9)	<0.0001	32.8 (26.2–38.6)	31.8 (26.7–37.5)	0.859
<40		165 (67.3%)	80 (83.3%)	0.003	30 (83.3%)	50 (83.3%)	1.000
MCV, fl	82–100	90.4 (86.7–93.9)	90.5 (87.7–93.9)	0.753	90.4 (87.4–94.6)	90.6 (87.7–93.3)	0.976
<82		16 (6.5%)	7 (7.3%)	0.801	3 (8.3%)	4 (6.7%)	1.000
MCH, pg	27–34	30.9 (29.5–31.9)	30.3 (29.1–31.4)	0.029	30.2 (28.9–31.5)	30.4 (29.1–31.4)	0.765
<27		13 (5.3%)	8 (8.3%)	0.296	3 (8.3%)	5 (8.3%)	1.000
MCHC, g/L	316–354	338.0 (328.0–348.0)	321.0 (305.0–337.8)	<0.0001	320.0 (303.5–336.8)	321.0 (305.0–338.5)	0.934
<316		22 (9.0%)	39 (40.6%)	<0.0001	15 (41.7%)	24 (40.0%)	0.872
RDW, %	11.5–14.5	14.1 (11.6–18.1)	15.5 (11.8–19.4)	0.017	15.6 (11.7–19.1)	15.5 (12.1–19.5)	0.806
>14.5		152 (62.0%)	53 (55.2%)	0.247	21 (58.3%)	32 (53.3%)	0.633

For COVID-19 patients admitted at Yangzhou, China in August 2021 (Table 2), the epidemiological investigation confirmed that their infections were due to the spreading of delta variant SARS-CoV-2 (6). Their hematological profiles were divided into groups of patients with no, partial (one-dose), and full (two-dose) vaccinations, and then unvaccinated delta variant COVID-19 patients in Yangzhou were compared to (unvaccinated) patients in Wuhan as well as the partially or fully vaccinated patients, respectively. Anemic conditions were ameliorated in delta variant SARS-CoV-2 infection, as RBC counts, hemoglobin, and hematocrit were substantially improved and the corresponding patient ratios with the abnormal values of the above parameters became significantly reduced. Other RBC indices showed a slight difference from those in COVID-19 patients in Wuhan. Notably, RDW exhibited markedly lowered values in delta variant infected patients at Yangzhou, where only a marginal portion (4.2%) of patients showed abnormally high values. Partial vaccination did not alleviate the anemia, but fully vaccinated patients demonstrated higher RBC counts and lower RDWs with significance, albeit the patient ratios with deranged hematological data showed no difference from those without vaccination.

**Table 2:** Baseline hematological data were compared between unvaccinated COVID-19 patients infected by wild-type and delta variant SARS-CoV-2 (exhibited by *p*^0^ values) and between delta variant COVID-19 patients unvaccinated and partially vaccinated (exhibited by *p*^1^ values), and between delta variant COVID-19 patients unvaccinated and fully vaccinated patients (exhibited by *p*^2^ values).

	**Normal**	**p^0^**	**Wild type (*n* = 245)^**0**^**	**Delta variant (*n* = 120)**	**Partially vaccinated (*n* = 60)^**1**^**	**p^1^**	**Fully vaccinated (*n* = 156)^**2**^**	**p^2^**
RBCs, ×10^12^/L	4.3–5.8	0.014	4.3 (3.9–4.6)	4.4 (4.0–4.9)	4.6 (4.1–4.9)	0.180	4.5 (4.2–4.9)	0.026
<4.3		0.033	125 (51.0%)	47 (39.2%)	19 (31.7%)	0.325	48 (30.8%)	0.146
Hemoglobin, g/L	130–175	0.003	128.0 (115.0–140.0)	135.5 (122.0–143.8)	137.0 (125.3–151.3)	0.110	137.0 (126.0–148.8)	0.086
<130		0.028	132 (53.9%)	50 (41.7%)	23 (38.3%)	0.668	54 (34.6%)	0.231
HCT, %	40–50	0.005	37.5 (34.3–40.8)	39.4 (36.3–42.7)	39.7 (37.6–42.9)	0.138	40.1 (37.0–43.2)	0.104
<40		0.014	165 (67.3%)	65 (54.2%)	32 (53.3%)	0.916	76 (48.7%)	0.369
MCV, fl	82–100	0.028	90.4 (86.7–93.9)	89.2 (86.4–92.0)	88.7 (86.5–91.3)	0.667	88.2 (86.0–91.2)	0.122
<82		0.961	16 (6.5%)	8 (6.7%)	0 (0)	0.053	9 (5.8%)	0.759
MCH, pg	27–34	0.029	30.9 (29.5–31.9)	30.4 (29.3–31.3)	30.6 (30.0–31.6)	0.841	30.3 (29.2–31.2)	0.419
<27		0.835	13 (5.3%)	7 (5.8%)	0 (0)	0.097	8 (5.1%)	0.798
MCHC, g/L	316–354	0.102	338.0 (328.0–348.0)	340.0 (334.0–347.0)	342.0 (335.0–349.0)	0.437	339.5 (334.0–348.0)	0.855
<316		0.180	22 (9.0%)	6 (5.0%)	1 (1.7%)	0.427	3 (1.9%)	0.183
RDW, %	11.5–14.5	0.001	14.1 (11.6–18.1)	12.4 (12.0–13.2)	12.4 (11.9–13.0)	0.307	12.3 (11.8–12.7)	0.035
>14.5		<0.0001	120 (49.0%)	5 (4.2%)	1 (1.7%)	0.665	4 (2.6%)	0.509

## Discussion

This study is invalid to assess whether the deformed RBCs subject to COVID-19 infection were owing to the direct viral insults on erythrocytes or the indirect deterioration by triggered proinflammatory cytokines. Neither could our study validate if timely vaccinations protect patients from a blood infection. Of note, anemia in COVID-19 patients may partially result from iron deficiency in dietary supplements among Chinese adults, long before SARS-CoV-2 infection (11). Nevertheless, previous studies have revealed that elevated RDW in COVID-19 patients was related to the disease severity and mortality, especially in non-anemic patients (12). The result here suggested a lessened viremic effect of delta variant SARS-CoV-2 on RBCs, as the infected patients showed minimal alteration on RDW as an important index for COVID-19 hematological impact. Hence, unlike that in COVID-19 cases infected by wild-type SARS-CoV-2, RDW might not be a valid prognostic indicator of COVID-19 severity in delta variant SARS-CoV-2 infection, although this variant of concern still possesses increased virulence, transmissibility, and immune escape (13).

## Data Availability Statement

The raw data supporting the conclusions of this article will be made available by the authors, without undue reservation.

## Ethics Statement

The studies involving human participants were reviewed and approved by Research Ethics Commissions in the First People's Hospital of Jiangxia District (FPHJD) in Wuhan City of Hubei Province, China, and in the Third People's Hospital of Yangzhou City (TPHYC), Jiangsu Province, China. Written informed consent for participation was not required for this study in accordance with the national legislation and the institutional requirements.

## Author Contributions

JZ and ZT conceived the idea and designed the study. JZ, JH, SF, DD, and ZT contributed to the data acquisition, processing, and table preparation. XH and ZT contributed to the statistical analysis. All authors contributed to the manuscript writing and approved the manuscript submission.

## Conflict of Interest

The authors declare that the research was conducted in the absence of any commercial or financial relationships that could be construed as a potential conflict of interest.

## Publisher's Note

All claims expressed in this article are solely those of the authors and do not necessarily represent those of their affiliated organizations, or those of the publisher, the editors and the reviewers. Any product that may be evaluated in this article, or claim that may be made by its manufacturer, is not guaranteed or endorsed by the publisher.

## Abbreviations

COVID-19: Coronavirus disease 2019
ICU: intensive care unit
MCH: mean corpuscular hemoglobin
MCHC: mean corpuscular hemoglobin concentration
MCV: mean corpuscular volume
RBC: Red blood cell
RDW: Red blood cell distribution width
SARS-CoV-2: Severe acute respiratory syndrome coronavirus 2.
