# Supplementary material for: Association Between Red Blood Cell Distribution Width and COVID-19 Severity in Delta Variant SARS-CoV-2 Infection
Source: Front Med (Lausanne). 2022 Feb 21;9:837411. doi: 10.3389/fmed.2022.837411 (PMC8899032; doi:10.3389/fmed.2022.837411)
Supplement: Supplementary file 1 [file Data_Sheet_1.doc]

Supplementary Materials

for

**Association between red blood cell distribution** **width and COVID-19 severity in delta variant SARS-CoV-2 infection**

Jianguo Zhang^1,†^, Jianhui Hu^2,†^, Xing Huang^3^, Shixiang Fu^4^, Daoyin Ding^5^, Zhimin Tao^1, 6, *^

^1^Department of Emergency Medicine, The Affiliated Hospital of Jiangsu University, Zhenjiang, Jiangsu 212001, China.

^2^Department of Laboratory Medicine, Zhenjiang Hospital Affiliated to Nanjing University of Chinese Medicine, Zhenjiang Hospital of Traditional Chinese

Medicine, Jiangsu 212003, China.

^3^Center for Evidence-based and Translational Medicine, Zhongnan Hospital of Wuhan University, Wuhan 430071, China.

^4^Department of Hepatology, The Third People’s Hospital of Yangzhou City, Jiangsu 225002, China.

^5^Department of Critical Care Medicine, The First People’s Hospital of Jiangxia District, Wuhan, Hubei 430200, China.

^6^Jiangsu Province Key Laboratory of Medical Science and Laboratory Medicine, School of Medicine, Jiangsu University, Zhenjiang, Jiangsu 212013, China.

^†^Jianguo Zhang and Jianhui Hu contributed equally to this work.

*Correspondences should be addressed to:

Zhimin Tao: [jsutao@ujs.edu.cn](mailto:jsutao@ujs.edu.cn)

**METHODS**

**Patients**

In one cohort 341 COVID-19 patients were admitted at the First People’s Hospital of Jiangxia District (FPHJD) in Wuhan, Hubei Province, China, during January 2020 to April 2020. This cohort contains 96 patients in the intensive care unit (ICU) and 245 patients in non-ICU isolation ward. In the other cohort 336 COVID-19 patients infected by delta variant SARS-CoV-2 were hospitalized at the Third People’s Hospital of Yangzhou City (TPHYC), Jiangsu Province, China, in August 2021, where no ICU patients were reported. All COVID-19 patients were diagnosed and confirmed by following a standard procedure(Huang et al., 2020). Exclusion criteria were adopted as previously reported(Hu et al., 2022). The study was approved by the Research Ethics Commission of FPHJD and TPHYC, respectively. All patient information remains anonymous, and written consent was waived.

**Vaccinations**

For the cohort of patients infected by delta variant SARS-CoV-2 in Yangzhou, China, 35.7% was unvaccinated, 17.9% was partially vaccinated and 46.4% was fully vaccinated. Two types of inactivated vaccines (SinoVac or Sinopharm) were provided, where 18.2% patients were given Sinopharm vaccines, 20.2% were given Sinovac vaccines, and 26.2% were given uncertain vaccines (either Sinopharm or Sinovac). A dose of vaccine was counted effective only if the time between the vaccine shot and the disease onset was longer than 14 days. Patients were considered partially vaccinated if only one effective shot of vaccine was given or fully vaccinated if two effective shots of vaccines were completed.

**Statistical analysis**

The categorical variables were described as frequency rates and percentages, and continuous variables were applied to describe the median and quartile range (IQR) values. Comparison of continuous variables between two cohorts was analyzed with Mann-Whitney test. χ^2^ test was used to compare the proportion of categorical variables, and the Fisher exact test was employed when data were limited. All statistical analyses were performed using GraphPad Prism 5.0 software (GraphPad Software, Inc., San Diego, CA), and statistics analyses adopted published methods(Zhang et al., 2021a;Zhang et al., 2021b;Zhang et al., 2021c;Hu et al., 2022). A two-sided α of <0.05 was considered statistically significant.

**References**

Hu, Z., Huang, X., Zhang, J., Fu, S., Ding, D., and Tao, Z. (2022). Differences in Clinical Characteristics Between Delta Variant and Wild-Type SARS-CoV-2 Infected Patients. *Frontiers in Medicine* 8.

Huang, C., Wang, Y., Li, X., Ren, L., Zhao, J., Hu, Y., Zhang, L., Fan, G., Xu, J., Gu, X., Cheng, Z., Yu, T., Xia, J., Wei, Y., Wu, W., Xie, X., Yin, W., Li, H., Liu, M., Xiao, Y., Gao, H., Guo, L., Xie, J., Wang, G., Jiang, R., Gao, Z., Jin, Q., Wang, J., and Cao, B. (2020). Clinical features of patients infected with 2019 novel coronavirus in Wuhan, China. *Lancet* 395**,** 497-506.

Zhang, J., Ding, D., Huang, X., Zhang, J., Chen, D., Fu, P., Shi, Y., Xu, W., and Tao, Z. (2021a). Differentiation of COVID-19 from seasonal influenza: A multicenter comparative study. *J Med Virol* 93**,** 1512-1519.

Zhang, J., Huang, X., Ding, D., and Tao, Z. (2021b). Platelet-driven coagulopathy in COVID-19 patients: in comparison to seasonal influenza cases. *Exp Hematol Oncol* 10**,** 34.

Zhang, J., Huang, X., Ding, D., Zhang, J., Xu, L., Hu, Z., Xu, W., and Tao, Z. (2021c). Comparative Study of Acute Lung Injury in COVID-19 and Non-COVID-19 Patients. *Front Med (Lausanne)* 8**,** 666629.
